# Camptocormia and shuffling gait due to a novel *MT-TV* mutation: Diagnostic pitfalls

**DOI:** 10.1212/NXG.0000000000000147

**Published:** 2017-04-05

**Authors:** Jens Reimann, Diana Lehmann, Steven A. Hardy, Gavin Falkous, Charlotte V.Y. Knowles, Rachel L. Jones, Wolfram S. Kunz, Robert W. Taylor, Cornelia Kornblum

**Affiliations:** From the Department of Neurology (J.R., C.K.), Department of Epileptology (W.S.K.), Life and Brain Centre (W.S.K.), and Centre for Rare Diseases Bonn (ZSEB) (C.K.), University Hospital of Bonn, Germany; Department of Neurology (D.L.), University of Halle/S., Germany; and Wellcome Trust Centre for Mitochondrial Research (D.L., S.A.H., G.F., C.V.Y.K., R.L.J., R.W.T.), Institute of Neuroscience, The Medical School, Newcastle University, Newcastle upon Tyne, UK.

Camptocormia, the disabling flexion of the spine in upright, but not supine position, has been reported in a range of central nervous and neuromuscular conditions and is associated with aging, too. In many cases, e.g., Parkinson disease, further clinical symptoms will clarify its association, if not pathophysiology. In others, a tangle of signs and symptoms obscures the etiology. Here, we present a new solution to this challenging and complex clinical problem.

A 71-year-old Caucasian woman presented with a 3-year history of unstable, short-stepping slow, shuffling gait and complained of deteriorating handwriting. Her medical history included basalioma, malignant melanoma, hypothyreosis, bilateral cataract and hypoacusis, gastroesophageal reflux, prediabetes, and thoracal and lumbal disc herniation. While her mother and a brother suffer from type II diabetes and a sister has a thyroid disorder, no other recurrent, neuromuscular, or movement disorders are known in the family. A brother died in childhood of unclear causes, a further brother in adulthood of a liver condition. Her son, daughter, and 2 grandsons are well. Clinical examination revealed slow horizontal saccades. Deep tendon reflexes were brisk, but for diminished ankle reflexes. Babinski response was equivocal on the right. Muscle tone and bulk appeared normal, but there was weakness of proximal lower limb muscles and foot extensors (MRC 4). Steps were short and their number for 180° turn increased. Positive Romberg test and inability to tandem walking suggested sensory ataxia, but sensory examination was otherwise normal. At follow-up, a slightly stooped posture progressed into camptocormia (figure, A) without signs of dystonia or muscle rigidity. Mild weakness of proximal arm muscles was found. Spinal MRI showed cervical stenosis without signs of myelopathy, but fatty replacement of paraspinal muscles. Initial neurophysiologic examinations showed prolonged cortical latency after tibial nerve stimulation, but transcranial stimulation gave normal total and central motor conduction times, and EMG of the vastus lateralis muscle was normal. Repeated neurography revealed a mild sensorimotor polyneuropathy. Levodopa treatment and CSF drainage under the suspicion of Parkinson disease or normal pressure hydrocephalus (NPH) due to enlarged lateral ventricles in an otherwise unremarkable brain MRI and urge incontinence were unsuccessful. DAT scan, EEG, Holter ECG, blood pressure monitoring, orthostatic test, and CSF analysis, including biomarkers for neurodegeneration (beta-amyloid, tau, and phospho-tau), revealed no abnormalities. Mini-Mental State examination and Montreal Cognitive Assessment indicated mild cognitive impairment. Maximum serum creatine kinase was 236 U/L (<170 U/L). Genetic and antibody analysis ruled out facioscapulohumeral muscular dystrophy types 1 and 2 as well as myasthenic syndromes, respectively.

**Figure:**
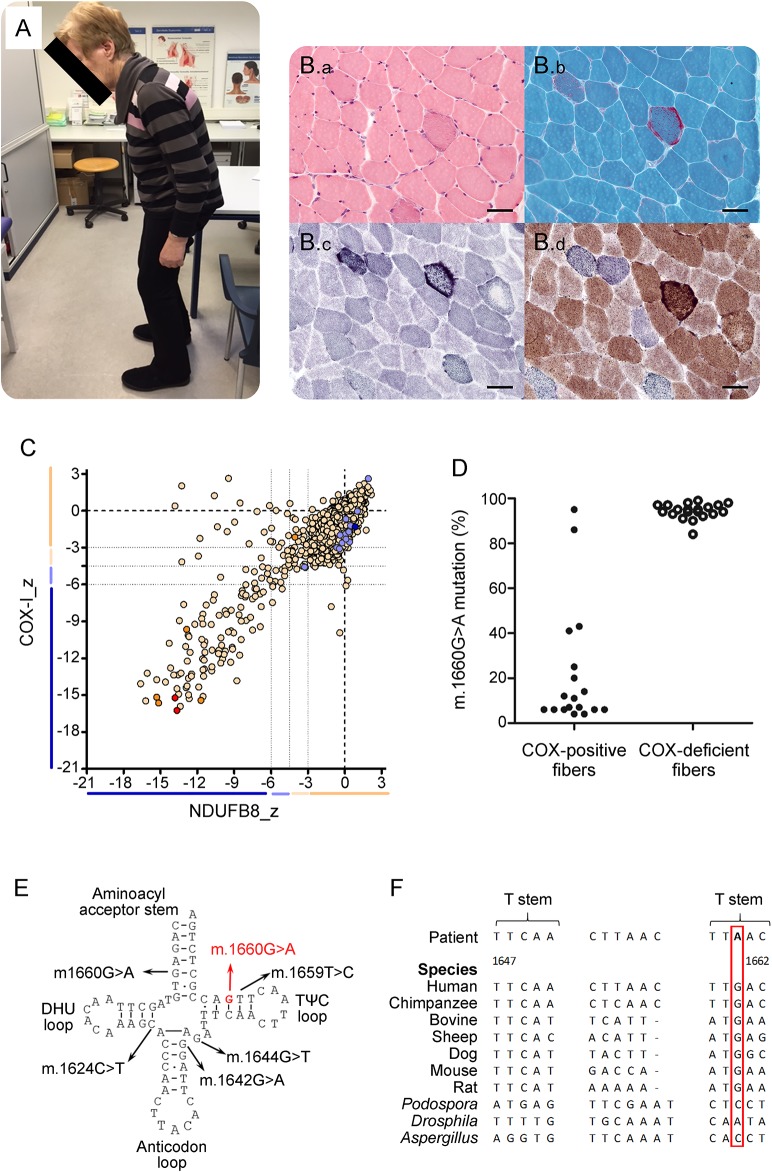
Clinical, histopathologic, and molecular genetic characterization of the m.1660G>A *MT-TV* mutation (A) Camptocormia as the main clinical feature of the patient harboring the novel 1660G>A *MT-TV* mutation. (B) Serial hematoxylin and eosin (B.a), modified Gomori trichrome staining (B.b), succinate dehydrogenase (SDH) (B.c), and cytochrome *c* oxidase (COX)-SDH histochemistry (B.d) showing ragged red fibers and COX-deficient fibers (scale bar = 50 μm). (C) Result of the quadruple OXPHOS immunofluorescence analysis, confirming the presence of fibers lacking both complex I (NDUFB8) and complex IV (COX-1) expressions. (D) Single muscle fiber mutation load segregation. The graph shows the mutation load measured in individual COX-positive (closed dots) and COX-deficient fibers (open dots) laser microdissected from the patient muscle biopsy. (E) Schematic representation of the cloverleaf structure of the mitochondrial (mt)-tRNA Val molecule and the corresponding location of the pathogenic mutation (marked in red) and previous reported mt-tRNA Val mutations (black). (F) Phylogenetic conservation of the appropriate regions of the mt-tRNA Val gene sequence for the m.1660G>A mutation.

Biceps brachii muscle biopsy showed angular atrophic fibers, a few degenerating fibers and increased lipofuscin deposition. Modified Gomori trichrome staining and oxidative enzyme reactions revealed ragged red fibers and ∼6% cytochrome *c* oxidase (COX)-deficient fibers (figure, B). Occasional ragged red fibers appeared COX positive. In skeletal muscle tissue homogenate, activities of mitochondrial respiratory chain complexes I and IV normalized against citrate synthase activity were mildly decreased to 0.07 (controls: 0.11 ± 0.03 [n = 11]) and 1.38 (2.7 ± 0.5 [n = 11]) U/g, respectively, while quadruple OXPHOS immunofluorescence^1^ confirmed the presence of fibers lacking both complex I (NDUFB8) and complex IV (COX-1) expression, confirming a multiple respiratory chain defect (figure, C). Mitochondrial DNA (mtDNA) sequencing revealed a previously unreported heteroplasmic m.1660G>A *MT-TV* variant present at highest levels in the muscle (35% mutation load), with lower levels in urinary epithelial sediments (13%) and blood (9%), consistent with the segregation pattern of a pathogenic mtDNA mutation. Single-fiber segregation studies clearly confirmed pathogenicity, showing a statistically significant higher m.1660G>A mutation load in COX-deficient fibers (94.30 ± 0.76 [n = 20]) than in COX-positive fibers (22.17 ± 6.49 [n = 18], *p* < 0.0001, unpaired *t* test) (figure, D).

Camptocormia has been reported in association with myopathic and mitochondrial defects^2–7^ with recent research suggesting limb muscle biopsy as a recommended diagnostic procedure.^7^ Here, we demonstrate a rare late-onset mitochondrial disorder due to a novel pathogenic *MT-TV* mutation (figure, E and F) mimicking much more common clinical conditions like NPH, subcortical artherosclerotic encephalopathy, or extrapyramidal movement disorders. Particularly, the coexistence of a shuffling gait, peripheral neuropathy, axial weakness, and bent spine at an advanced age may masquerade a mitochondrial pathophysiology and lead to erroneous diagnosis and treatment. Our finding adds to the spectrum of differential diagnostic considerations in gait and balance disorders in the elderly and underlines the importance of skeletal muscle biopsy as a major diagnostic tool in these patients. Mitochondrial disorders frequently lead to multisystemic disease and may manifest even in late adulthood where symptomatic treatment options and tailored clinical advice are of utmost importance for affected patients.
